# Quarterly Summary

**Published:** 1855-10

**Authors:** 


### QUARTERLY SUMMARY.

### CHEMISTRY.

1.—*Gastric Juice.*—Longet has published in the *Comptes Rendus* for February, an abstract of the results of examinations of the action of gastric juice upon the albuminoid aliments.

He finds that they are changed into albuminose, which greatly modifies Trommer and Banewill's tests for sugar. This modification was noticed by Daltin of New York, on whose paper we made some remarks in our editorial department for April, but he attributed it erroneously to the gastric juice. Longet finds that it is due to the albuminose. He proves this by various admixtures of albuminous matters with glucose, and of the albuminose resulting from digestion with the substance. In the former mixtures, a copious precipitate of red oxyd was thrown down; in the latter no precipitate took place but a beautiful violet coloration was observed.

In living animals the blood of the vena porta and that of the hepatic veins was examined, and in no case could these tests detect sugar in the former vessel, while its reaction was very distinct in the latter, as well as in the intestine and stomach.

An incident which occurred during the progress of these examinations is highly instructive, especially in reference to Bernard's famous theory of the glucoganic function of the liver. A rabbit kept fasting for 48 hours, ate some blood with which glucose had been mixed. The animal was killed three hours and three quarters after this singular repast. The abdomen was immediately opened, the vena porta tied and the blood examined. Trommer's test gave no precipitation in this blood, though it did in that of the right side of the heart and in the contents of the stomach, intestine and liver. Yet it is absolutely certain that it existed in the blood of the vena porta, for independent of the history which could leave no reasonable doubt, fermentation disclosed its presence in that fluid.

The bearing of these experiments upon Bernard's theory is very decided, since Banewill's liquor was the test for glucose, upon which he relied.

The failure to detect sugar in the stomach, after a meal of starch, depended in all probability upon the same cause.

2.—*Pancreatic Juice.*—Krœger has recently examined the pancreatic juice, and the results of his analyses differ materially from those of his predecessors. Thus, in regard to the proportion of solid matter, Bidder and Schmidt find 9.924. Frericks, 1.36, and Krœger 1.848 per cent. of solid matter. We can only infer from this that the fluid must vary much in its chemical constitution.

As to its *physiological action,* he confirms the results of the German experimental observer. He finds that one part of this fluid will transform in half an hour, at a temperature of 35° centigrade, 4.672 parts of starch into sugar. This, however, can be by no means its chief function, since, according to Frerich's experiments, it requires but 490 grammes of starch to be transformed into sugar, in order to supply the daily waste of carbon by an adult man; which would require no less than 105 grammes of pancreatic juice, whereas the amount actually secreted is more than 5000 grammes.

It is therefore probable that this fluid may promote the continual interchange of liquids in the system. A particular connection appears to exist between it and the secretion of the stomach, the hydrochloric acid of the gastric juice separated by the glands of the stomach from the chloride of sodium, uses the soda of the pancreatic fluid to reproduce that salt. In favor of this theory, Krœger observes that the hydrochloric acid secreted for each kilogramme in weight of the dog in 24 hours amounts to 0.305 grammes, while that of the soda contained in the pancreatic juice secreted in the same time is 0.237 grammes, very nearly the equivalent of the acid, which is 0.259, an error quite within the limit of errors of analysis.

Berthelot has also been engaged in experiments on this fluid which he has published in the *Comptes Rendus.*

He examined its action upon lard and butter. Bare morrobutyrine was taken as an example of the latter, and under the influence of pancreatic juice, it was decomposed almost entirely into butyric acid and glycerine. Lard was also decomposed with the regeneration of a solid, crystallizable fatty acid, fusible at 61° c. Comparing these results with the action of saliva, it was found that the latter fluid produced no sensible change in butter.

3.—*Digestion of Starch.*—Blendlot is out with a memoir on this difficult subject in the *Annales de Chimie et de Physique.* He broaches some opinions which strike at the very foundation of our ideas of starch.

He undertakes to show in the first place, that the membrane investing the minute granules is nitrogenous. He proves the existence of nitrogen by heating starch and caustic potassa in a test tube, so that the vapor raising from them shall pass over a slip of reddened litmus paper, which is rendered blue by the ammonia, generated in consequence of the decomposition of the azotized membrane. He further asserts that the blue color produced with starch by free iodine proceeds not from the fecula itself, but from this nitrogenous matter, and cites the example of inulina which resembles fecula in every respect but this.

He also shows that the granules of starch are not destroyed by a heat of 302° F. and that they remain intact even after pacification, or the formation of bread.

He reaffirms the exploded proposition that saliva cannot metamorphose starch into dextrine in an acid fluid, and quotes Mialhe to prove that on crude fecula, saliva exerts no such saccharifying influence. He infers that no change takes place in consequence of the admixture of pancreatic juice, and denies the possibility of any such transformation elsewhere than in the stomach.

He believes that the gastric juice disintegrates the granules by digesting their nitrogenous envelop. On experimenting upon the contents of the stomachs of animals after anylaceous meals, he found that there were some granules which still gave the blue colors with iodine, while there were others which that reagent only made yellow. In the upper part of the small intestines, only the latter class of granules was discovered. In the large intestine, their number gradually diminished, till in the rectum scarcely any could be found. The digestion of the azotized membrane having set the granules free, they are sufficiently minute to enter the chyliferous vessels along with the fat globules which penetrate the system by the same route.

4.—*Goitre.*—The question of the origin of this disfiguring disease has, as our readers well know, been long involved in obscurity. It is known to prevail in certain localities, as the gorges of the Alps and the valley of Derbyshire. In our own country, it is also common in parts of the coal formation on the western slopes of the Alleghanies, which resemble in their geological character the English site of the disease.

It has been supposed by some, that some positively deleterious agent, as magnesia, might produce it; or that the absence of iodine from the waters of a district was capable of bringing about this result. There are objections to both these theories.

Mr. E. Maumené has put forth another view; he suggests that the fluorides may be the malignant agents in this case. The mere fact that these salts have not been detected in the water of countries in which this disease is inclusive, amounts to nothing, for all chemists admit the extreme difficulty of detecting these salts.

To determine this point, M. Maumené took a dog and fed him on food mixed first with fluoride of calcium and then with a normal solution of fluoride of sodium. The animal's general health did not appear to suffer, but after a few months an enlargement of the neck, in the form of a collar, was observable. This the experimenter regarded as a goitre, and being unable to make confirmatory experiments, has published the results of this single case, which at least should serve to stimulate others to inquiry, and which may point out the proper course for them to adopt.

5.—*Tobacco Smoking.*—M. Malapert has been engaged in a series of researches, to determine the quantity of *nicotina* inhaled by smokers. He arranged an apparatus for burning tobacco in a continuous current of air and for arresting the products of the combustion. He discovered that 18 per cent. of the tobacco remained behind as ashes, the rest being consumed and passing off as vapor. The nicotine amounted to between 9 and 10 per cent. of the whole weight of the tobacco, as the boiling point of this poisonous, volatile alkaloid is high, most of it condenses in the segar or the tobacco of the pipe during the first part of the combustion. As the segar is gradually consumed, the fire reaches this condensed portion and volatilizes it again. Hence the difference between the beginning and the end of a pipe or segar. Hence also the disgusting smell of a half-smoked segar.

The volatilization of nicotine is promoted by the presence of moisture, which accounts for the fact that smokers are more easily sickened by moist than by thoroughly dried tobacco. A *new* pipe also interferes with the smoker because the clay absorbs the nicotine till it is saturated, and thereby diminishes the narcotic effect of the tobacco.

The *hookah* and other smoking apparatus of the orientals owe their harmlessness to their construction, which causes the condensation of nearly all the nicotine, &c., for it reaches the mouth of the smoker, since it must traverse a layer of water and expand in the chamber above.

As the result of his researches, M. Malapert advises smokers, 1st, to avoid moist tobacco; 2nd, to use only those pipes furnished with a condensing chamber; 3d, not to smoke either a pipe or segar after half the tobacco is consumed.

In the Chemist for June, Mr. Thornton J. Herapath has a paper on the subject of nicotine in the article of tobacco smokers, which forms a suitable *pendant* to the above.

Having voided his urine upon a heap of unslacked lime, after having smoked for several hours in succession, he noticed that the steam which was given off smelt strongly of tobacco. This attracted his attention, and he determined to collect some of his urine and subject it to analysis, in order to ascertain whether it contained nicotine. A couple of quarts of urine were mixed with a little dilute sulphuric acid and evaporated to the consistence of a thin syrup, dilute sulphuric acid being added from time to time, to neutralize the ammonia formed by the decomposition of the urea. It was then distilled, and the auro-chloride and platino-chloride of nicotine obtained from the distillate.

Mr. Herapath gives figures of these salts, as crystallized slowly or hastily. He applied the same process to the examination of other organic liquids and considers capable of being used for medico-legal purposes.

6.—*Sugar in the Liver.*—M. Figuier, recently read a paper on the subject of the glucogenic function of the liver, which is an important contribution to physiology. It will be remembered, that Bernard was the first to point out the existence of glucose or grape sugar in this organ, and he inferred from his experiments that the liver actually formed the sugar from the materials presented to it by the portal blood, and that independently of all vegetable food.

In order to determine the question of this formative power of the liver, it is of course necessary to decide upon the presence of glucose in the blood. This had never been detected up to the time of Bernard's experiments, but M. Figuier, thinking that the failure to recognize it might depend on the methods of analysis, determined to adopt a new plan. He defibrinated fresh blood in the usual manner, and then precipitated the albumen with the corpuscles entangled in it by means of alcohol. This gave a colorless solution, which, on evaporation to dryness and digestion in distilled water, indicated sugar by Tromm's test. This was a uniform result of all their experiments. The blood from a slaughter house yielded alcohol and carbonic acid, by mixture with yeast, showing conclusively the presence of sugar in it.

A quantitative examination showed that the blood of the rabbit contained 0.57 per cent. of glucose, while the liver of the same animal contained only 1 per cent. In ox blood the proportion was 0.48 and in the blood of man 0.58 per cent.

Now, as the liver contained only about double the quantity of sugar found in the blood, it follows that that organ cannot be considered glucosive. Being a depository again, however, it contains more glucose than the blood, just as a filter that has not been washed will contain a larger proportion of solid matter than the liquid which ran through it. The same consideration will explain the increased quantity of sugar in this organ immediately after food has been taken, or during the process of digestion. The presence of sugar in the livers of purely carnivorous animals is also deprived of its significance, since the blood in the vessels of the flesh they ate contained the glucose.

The sugar, therefore, must come from without, from the food, and the increase of the secretion upon irritation of the medulla oblongata seems explicable on the hypothesis of an interference with the respiratory act, which allowed the glucose to accumulate in the blood.

The *Comptes Rendus* for March 12th, contains a paper by Lehmaun upon the same subjects. The test employed was fermentation.

According to this chemist, the blood of the vena porta never contains the least traces of sugar in days of fasting or when fed on meat. When fed with cooked potatoes their portal blood contained sugar, but in so small quantity that it could not be estimated. The blood of the hepatic veins always contains a large proportion of sugar, and a table given by the author expresses this difference in the saccharine contents of the two vessels in a very marked manner.

He also states, that the blood of the portal vein contains fibrine, whereas, that of the hepatic veins does not, the slight coagulum, which is formed on whipping that fluid, consisting almost exclusively of white corpuscles. A large quantity of fibrine, therefore, disappears in the liver, and the same is true of albumen. Fat also is more abundant in the portal than in the hepatic veins, and the globules of the former blood contain more iron and less globuline extractive matter, salts than those of the latter. Hœmatin consequently is absorbed by the liver, a fact which goes to establish the view advanced in the author's work, on physiological chemistry, that the hœmatin of the globules is used to form the bile-pigment.

The comparative analyses of the blood of different vessels, show that that of the hepatic veins contains the most sugar, after them the blood of the vena-cava inferior being the richest in saccharine matter. After the blood has traversed the lungs and entered the arteries, sugar is no longer present in it, unless the veins have contained an abnormal amount of glucose. He regards his results as confirming Bernard's theory of the double function of the liver.

He also ascertained that he could generate sugar from hœmatosine, and he believes he has formed it from hæmatine.

This paper of Lehmaun's brought out another from Figuier, published in the *Comptes Rendus* for March 26th. His article contains the history of some experiments to decide the question of the glucogenic function of the liver. A young dog having been kept fasting for three days was fed for 8 days with raw beef, kept fasting again for 40 hours, and then fed with two pounds and a half of raw beef. Two hours after this the abdomen was opened, and the blood taken from the vena porta, the mesenteric veins, the inferior cava and the right ventricle of the heart.

The blood of the vena porta, and of the mesenteric veins was found to contain the sugar, the former in the proportion of 0.248 per cent. The blood above the liver contained only traces of glucose. In another experiment, the blood of the portal vein contained 0.231, and that above the liver 0.304, per cent. of glucose.

M. Figuier regards the liver as a reservoir of sugar, which remains in that organ for a time, and is then taken into the circulation through the hepatic veins. Digestion being completed, and all the saccharine matter absorbed from the alimentary tube, the blood returning through the vena porta, contains no glucose, but, in passing through the liver takes up a fresh quantity of this substance, and carries it through the hepatic veins into the general circulation.

He insists that Lehmaun's paper does not controvert his results, as that chemist experimented upon fasting animals, in which, according to Figuier's own investigations, sugar ought to disappear from the portal vein.

In April 16th, appeared two papers on the other side of the question, one by Possiole, the other by Leconte, who assisted Bernard in his original experiments.

The former writer experimented on bitches, feeding the animals on various substances, and examining their milk quantitatively for sugar, under the different systems of diet, as well as during absolute fasting. We give his results.

1st. Sugar may be formed in the animal economy at the expense of the nitrogenous aliment, and perhaps of the fatty bodies.

2d. Alimentation or fat alone does not appear to diminish the proportion of sugar in the organism.

3d. The amylaceous aliments are transformed into sugar by the digestive action.

4th. When animals are fed upon amylaceous matter, the blood of the vena porta contains a considerable proportion of sugar.

5th. In animals fed upon meat, no sugar exists in the vena porta, it is found, on the contrary, in notable quantity in the hepatic veins, in the vena cava inferior and the arterial blood.

6th. The blood of the vena porta contains no sugar when the animal has been enduring complete abstinence from food.

7th. Consequently, we are obliged to admit, that in animals fed upon nitrogenous matters and fat, the production of sugar takes place in the liver.

Leconte's paper records the results of experiments conducted according to Figuier's plan of operating upon an alcoholic extract. He positively denies the presence of sugar in the vena porta of animals fed on meat, and as positively affirms its existence in the vena cava, and the hepatic veins. He also concludes that the sugar is formed, not merely condensed in the liver, and finds that the blood of the hepatic veins contains a larger per centage of solid matter than that of the vena porta.

Finally, on June 18th, the same journal contains a report from the committee appointed by the Academy to inquire into this subject. The names of Pelouze, Rayer and Dumas, who composed this investigating committee, are sufficient guarantee for the care with which their researches were conducted. In the two questions of fact involved in the controversy, viz. the absence of sugar in the portal, and its presence in the hepatic blood of animals feeding on meat alone, they decide in favor of Bernard. The further questions of the production of sugar by the liver, and the source whence that organ obtains it, they leave open.

We must, therefore, for the present, receive this theory of Bernard's as a substantial and important advance in the science of physiology.

